# Spatial analysis of American cutaneous leishmaniasis in the state of Amazonas

**DOI:** 10.11606/s1518-8787.2024058005662

**Published:** 2024-03-27

**Authors:** Mirely Ferreira dos Santos, Camila Lorenz, Francisco Chiaravalotti-Neto, Tamara Nunes Lima-Camara

**Affiliations:** I Universidade de São Paulo Faculdade de Saúde Pública Programa de Pós-Graduação em Saúde Pública São Paulo SP Brasil Universidade de São Paulo. Faculdade de Saúde Pública. Programa de Pós-Graduação em Saúde Pública. São Paulo, SP, Brasil; II Universidade de São Paulo Instituto de Estudos Avançados São Paulo SP Brasil Universidade de São Paulo. Instituto de Estudos Avançados. São Paulo, SP, Brasil; III Universidade de São Paulo Faculdade de Saúde Pública Departamento de Epidemiologia São Paulo SP Brasil Universidade de São Paulo. Faculdade de Saúde Pública. Departamento de Epidemiologia. São Paulo, SP, Brasil

**Keywords:** Leishmaniasis, Cutaneous, Epidemiological Monitoring, Geographic Information Systems, Spatial Analysis

## Abstract

**OBJECTIVE:**

To evaluate, using spatial analysis, the occurrence of American Cutaneous Leishmaniasis (ACL) and analyze its association with the municipal human development index (MHDI) and deforestation in the state of Amazonas, Brazil, from 2016 to 2020.

**METHODS:**

This ecological study, carried out from January 2016 to December 2020, included the 62 municipalities of the state of Amazonas. The incidence rate of ACL was determined in space and time. Using Multiple Linear Regression by Ordinary Least Squares (OLS) and Spatial Autoregressive Regression (SAR) models, the relationship between incidence rates and Human Development Index (HDI) and deforestation was analyzed., The high- and low-risk clusters were identified by employing the Getis-Ord Gi* statistic.

**RESULTS:**

A total of 7,499 cases of ACL were registered in all 62 municipalities in the state. Most cases were in male (n=5,924; 79.24%), with the greatest frequency in the population aged from 20 to 39 years (n=3,356; 44.7%). The incidence rate in the state of Amazonas was 7.34 cases per 100,000 inhabitants-year, with the municipalities of Rio Preto da Eva and Presidente Figueiredo showing the highest rates (1,377.5 and 817.5 cases per 100,000 population-year, respectively). The ACL cases were clustered into specific areas related to those municipalities with the highest incidence rates. The SAR model revealed a positive relationship between ACL and deforestation.

**CONCLUSIONS:**

The occurrence of ACL was evident in a variety of patterns in the state of Amazonas; the high incidence rates and persistence of this disease in this state were linked to deforestation. The temporal distribution showed variations in the incidence rates during each year. Our results can help optimize the measures needed to prevent and control this disease in the state.

## INTRODUCTION

American Cutaneous Leishmaniasis (ACL) ranks among the main public health issues in 85 countries and across four continents (the Americas, Europe, Africa, and Asia), registering from 0.7 to 1.3 million new cases per year^1^. From a global perspective, ACL is one of the six most well recognized infectious diseases, as it has a high detection coefficient and causes physical deformities^1^. For this reason, the World Health Organization (WHO) classified ACL in 2014 as an emerging and uncontrolled Neglected Tropical Disease (NTD) and, unfortunately, it has retained this status to this day^2^.

ACL is produced by a flagellate protozoan belonging to genus *Leishmania* spp., and the female sandfly (Diptera: Psychodidae, Phlebotominae) is the vector that transmits it to humans by its bite. The skin and mucous membranes of humans are affected^3^. Brazil alone is host to seven species of *Leishmania*, six of which belong to the *Viannia* subgenus and one to the *Leishmania* subgenus. There are three main species that cause ACL in humans, namely *Leishmania* (*Leishmania*) *amazonensis*; *Leishmania* (*Viannia*) *guyanensis*, and *Leishmania* (*Viannia*) *braziliensis*. The chief vector among the sandfly species occurring in Brazil are *Lutzomyia whitmani* (*Nyssomyia whitmani*), *Lu. intermedia* (*Nyssomyia intermedia*), *Lu. umbratilis* (*Nyssomyia umbratilis*), *Lu. wellcomei* (*Psychodopygus wellcomei*), *Lu. flaviscutellata* (*Bichromomyia flaviscutellata*), and *Lu. migonei* (*Migonemyia migonei*)^4^.

In Brazil, based on data extracted from the Department of Informatics of the Unified Health System (DATASUS), the ACL is distributed throughout the country. In 2019, Brazil recorded 15,484 new cases of ACL, revealing a detection coefficient of 7.37 cases per 100,000 inhabitants-year. In 2020, there was a rise in cases to 16,432 cases, but in 2021, the number of cases dropped to 15,023. Although the disease occurs throughout Brazil, in 2020, the highest percentage of cases was reported in the North region (44.5%), followed by the Northeast (17.4%), Mid-west (14.9%), Southeast (13%), and South (1.16%) regions of the country^5^.

In 2020, the COVID-19 pandemic was found to have directly affected surveillance and control measures for various diseases, ACL included. The pandemic has starkly influenced active search efforts, early detection, and patient treatment, besides other field activities. For instance, the state of Amazonas registered 1,297 cases of ACL in 2019, whereas in 2020, 1,663 cases were identified, i.e., revealing a 22% rise in cases compared to 2019^6^. In fact, the state of Amazonas ranked second in 2020, with the highest number of notifications in the northern region, after Pará (2,997), followed by the other states in the northern region, namely Acre (948), Rondônia (724), Amapá (462), Tocantins (423), and Roraima (195)^6^.

From some studies, it is evident that the crucial risk factors for the occurrence of ACL include the complex aspect of poverty and its close association with social inequality and vulnerability, as well as human development indices^7^. Furthermore, deforestation is closely related to all environmental changes, and can therefore induce changes in the habitat and breeding grounds of the sandfly vectors. To cite an example, a study carried out in the state of Amazonas found that new mining sites, the start of oil exploration, and the construction of related pipelines were strong factors that led to deforestation and increased contact between these workers and the sandfly vectors of ACL^10^.

The range of etiological agencies, reservoir hosts, and sandfly vectors, plus the current inadequate knowledge regarding some of the transmission dynamics, adds to the complexity of the ACL eco-epidemiology and the difficulty of controlling it^4^. A broader knowledge of ACL cases, as well as their spatial distribution, is vital for promoting more accurate and improved interventions since no specific vaccine is yet available as a prophylactic measure for reducing and controlling the number of cases of this disease^8^.

Spatial analysis tools facilitate the identification of the main sites that can be developed for more intensely targeted measures of leishmaniasis surveillance and control actions^11,12^. Thus, the goals of this study are (a) to evaluate the occurrence of American Cutaneous Leishmaniasis (ACL) using spatial analysis and (b) to analyze its association with the municipal human development index (MHDI) and deforestation in the state of Amazonas, Brazil, from 2016 to 2020.

## METHODS

### Study Design

This study is a descriptive and ecological investigation in which secondary data was employed to analyze all the municipalities in the state of Amazonas. Approval for this research was granted by the Research Ethics Committee of the School of Public Health of the University of São Paulo (CAAE: 31703620.9.0000.5421, number: 4.031.361).

### Study Area

**Figure 1:**
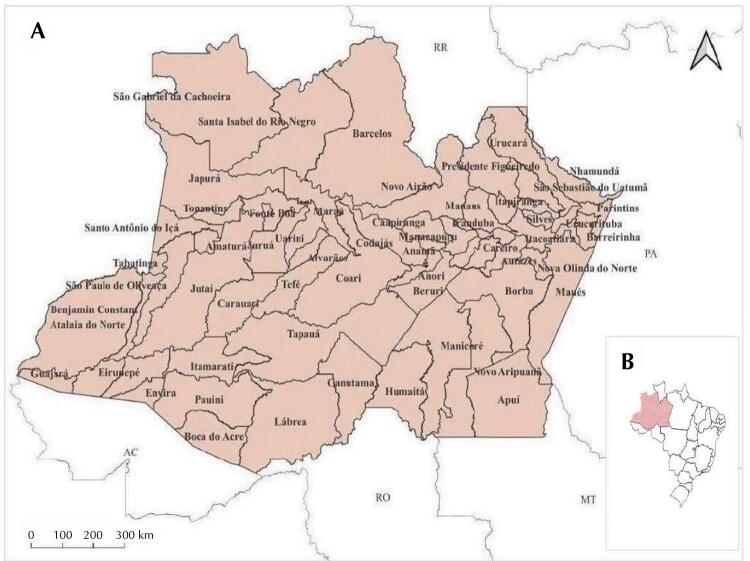
Study area. (A) State of Amazonas with the distribution of the municipalities and neighboring states (Acre, Rondônia, Mato Grosso, Pará, and Roraima). (B) Location of Amazonas, in the northern region of Brazil.

The state of Amazonas, in Brazil, occupies the northern region and extends over an area of 1,559,161.878 km^2^, bordering the state of Roraima to the north, Pará to the east, Mato Grosso to the southeast, Rondônia to the south, and Acre to the southwest. Based on the criteria of the Brazilian Institute of Geography and Statistics (IBGE), the state has been geographically divided into 62 municipalities (Figure 1).

In 2021, the state of Amazonas recorded a population of 4,269,995 inhabitants, making it the second largest in the northern region and equivalent to approximately 1.8 % Brazil’s current population^13^. The average Human Development Index (HDI) for the state of Amazonas is 0.674 (medium level). The capital, Manaus, is the largest city in the northern region, with 2.1 million inhabitants^13^.

### Case Definition and Inclusion/Exclusion Criteria

The study population included the autochthonous cases of ACL, reported from January 1, 2016, to December 31, 2020, in residents of the municipalities of the state of Amazonas. Autochthonous cases of ACL were defined as cases with the presence of a skin ulcer, with a granular bottom, and infiltrated edges in a frame, or with the presence of an ulcer on the nasal mucosa, with or without perforation or loss of the nasal septum, which may reach the lips and mouth (palate and nasopharynx), confirmed by laboratory diagnosis^1^.

The inclusion criteria were all new laboratory-confirmed cases of ACL reported in residents of municipalities of the state of Amazonas from January 1, 2016, to December 31, 2020. The exclusion criteria were all suspected cases with a negative laboratory diagnosis or with a confirmed laboratory diagnosis for another disease, as well as duplicate records.

### Study Variables and Data Collection

The incidence of the ACL was used as the response variable, after being transformed into a log to resolve outliers and bring it closer to the normal distribution. The explanatory variables used were the Municipal Human Development Index (MHDI) and deforestation. The log of the deforested area was also used, as it better explains the log of the incidence and resolves outliers. The other variables analyzed in this study were sex, age, color, area of residence (urban or rural), year, and municipality.

To calculate the incidence of the clusters during each year, we used the number of confirmed cases of ACL per municipality as the numerator and divided it by the denominator, which was the municipal resident population during the given period, and multiplied it by 100,000. To calculate the incidence for the analysis of the association between the variables over the entire study period, we used the mid-period population (year 2018).

Data regarding the patients with ACL were taken from the Information System for Notifiable Diseases (SINAN), with due permission from the Health Surveillance Foundation of the Amazonas state. Details regarding the population of the municipalities, demographic data, maps, and information about state highways were taken from the IBGE website^13^. The MHDI statistics were available in the United Nations Development Program (UNDP) database from the 2010 census.

The MHDI is a reliable indicator of a city’s degree of development^7^. It is determined as the average of the life expectancy index, education index, and income index or Gross Domestic Product (GDP), in the range 0 to 17. The closer the HDI value is to 1, the better are the population’s living conditions, health, education, and income^7^.

The National Institute for Space Research (INPE) provided environmental data on deforestation and deforested areas from 2016 to 2020, obtained by satellite monitoring of the Amazon Forest – Project for Monitoring Deforestation in the Legal Amazon by Satellite (PRODES).

The ACL incidence rates were assessed for the state of Amazonas, during the study period and by year, and for each municipality throughout the study period and each year. These rates were determined annually and standardized by sex and age category, keeping the population of the state of Amazonas in 2018 as the standard. For sex and age, direct standardization was used.

### Data Analysis

Before carrying out multiple linear regression modeling to analyze the relationship between incidence rates and HDI, as well as deforestation during the course of this study, an analysis was also carried out to investigate whether there were outliers and whether there was collinearity among the covariates. The log of the incidence rate (transformation to correct the outliers) was considered a dependent variable, whereas the HDI and the log of deforestation (transformation to correct outliers), which were not collinear, were considered independent variables. When checking for spatial autocorrelation, models with global spatial effects were used, which were designed to identify this correlation structure in only one parameter, which was then added to the linear regression model^14^.

The modeling began with the adoption of linear regression using the Ordinary Least Squares (OLS). Next, the Lagrange Multiplier (LM) test was used to evaluate the suitability of using Spatial Autoregressive Models (SAR): Spatial Lag Model (LAG) or Spatial Error Model (ERR). The models were compared in relation to the Akaike Information Criterion (AIC) values. This criterion indicated that the best model would be the one with the lowest AIC value^15,16^. To verify the assumptions of the regression model, the homogeneity of the residuals was determined using the plot at the levels of dispersion, normality, and spatial autocorrelation, assessed using the Moran’s I test.

The clusters with the high and low risk of ACL for each year of the study were subjected to Getis-Ord Gi* spatial analysis. With this method, local spatial associations were identified, considering a neighborhood matrix between the municipalities. This statistical tool, provided by Getis and Ord, is an indicator of local spatial association^17^. Using the Gi* statistic, the highest occurrence of the event/phenomenon, when the values are high, can be identified; however, low values suggest groupings of reduced occurrences^18^. Using the False Discovery Rate (FDR), the significance levels of statistical tests can be corrected for multiple comparisons^19^.

The R Studio software, version 1.3.1093, was used to prepare and analyze the databases, population estimates, calculations of standardized incidence rates, as well as all the choropleth maps, tables, and graphs.

## RESULTS

During the study period, 7,499 cases of ACL were verified and analyzed in the 62 municipalities of the state of Amazonas. Regarding distribution of cases by gender, from 2016 to 2020, the highest number appeared in males (79.24 %); in relation to age, the highest proportion was found in those aged from 20 to 39 (45.27 %); mixed ethnicity indicates the highest occurrence (80.57 %); and, in terms of places of residence, rural areas showed the greatest frequency of the disease (45.47%).

**Figure 2:**
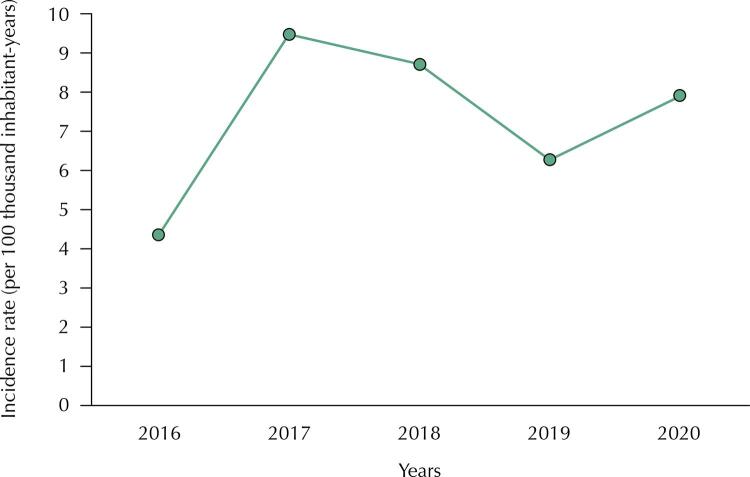
Incidence of American Cutaneous Leishmaniasis. Incidence rates of American Cutaneous Leishmaniasis per year in the state of Amazonas, Brazil, from 2016 to 2020.

The state of Amazonas recorded an average ACL incidence rate of 7,34 cases per 100,000 residents-year, during the study period. The rates showed variations in the rise and fall of the numbers for each year, with the lowest recorded in 2016 (4.34 cases per 100,000 inhabitants-year), and rising in 2017 (9.48 cases per 100,000 inhabitants-year), and a marginal decrease in 2018 (8.72 cases per 100,000 inhabitants-year). In 2019 (6.26 cases per 100,000 inhabitants-year), the incidence rate revealed a comparative decrease when in relation to the 2017 and 2018 values; however, a marginal rise was observed in 2020 (7.9 cases per 100,000 inhabitants-year) (Figure 2). All 62 municipalities in the state of Amazonas reported patients with ACL during the study period.

The municipalities with the highest incidence rates during the study period were Rio Preto da Eva, Presidente Figueiredo, and Silves, located near the metropolitan area of Manaus, the capital; followed by Envira, Boca do Acre, and Pauini, situated in the southern portion of the state. The lowest incidence rates were recorded in the municipalities of Urucurituba, Manaquiri, Codajás, Careiro da Várzea, and Anori, close to the capital.

In 2017, the municipality of Presidente Figueiredo recorded its highest values for the incidence rate (772.9 cases per 100,000 inhabitants-year), as well as in 2018 (817.5 cases per 100,000 inhabitants-year); however, in 2019, a substantial decline was evidenced (485.13 cases per 100,000 inhabitants-year), followed by a rise in 2020 (739.4 cases per 100,000 inhabitants-year). The highest incidence rates in the municipality of Rio Preto da Eva were also recorded in 2017 (1377.5 cases per 100,000 inhabitants-year) and 2018 (881 cases per 100,000 inhabitants-year); however, the numbers decreased in 2019 (386.84 cases per 100,000 inhabitants-year). In 2020, there was another increase (835.63 cases per 100,000 inhabitants-year). In the municipality of Silves, the highest incidence rates were recorded in 2017 (221.02 cases per 100,000 inhabitants-year) and again in 2020 (335.9 cases per 100,000 inhabitants-year).

The municipality of Envira registered the highest incidence rates in 2019 (204.7 cases per 100,000 inhabitants-year) and in 2020 (201.05 cases per 100,000 inhabitants-year). Boca do Acre, however, recorded variations with a rise and fall in incidence, with the highest rates observed in 2018 (173.7 cases per 100,000 inhabitants-year) and 2020 (207.9 cases per 100,000 inhabitants-year). The municipality of Pauini showed the highest incidence rates in 2017 (166.41 cases per 100,000 inhabitants-year) and 2018 (155.21 cases per 100,000 inhabitants-year).

From the results listed in the Table, the OLS models and spatial error model reveal spatial autocorrelation of the residuals, evaluated by Moran’s I, not meeting the requirement of independence of the errors. The outcome of the Lagrange Multiplier test indicated that the spatial regression model best suited to this data was the spatial error model. In the analysis of the residues of this model, Moran’s I (I = 0.04, p = 0.255) revealed the absence of spatial dependence, the residues showed zero mean, normality, and homoscedasticity. The spatial model was shown to be a better fit than the OLS model for the current data (showing a lower AIC).

**Table:** Incidence of American Cutaneous Leishmaniasis (ACL), Municipal Human Development Index (MHDI), and deforestation. Multiple linear regression models (Ordinary Least Square and spatial error) of the incidence rates of American Cutaneous Leishmaniasis (on a logarithmic scale) as a function of the Municipal Human Development Index and deforestation (on a logarithmic scale), in the state of Amazonas, Brazil, from 2016 to 2020.

Covariate	OLS model		Spatial error model	
	
	Coefficient	p-value	Coefficient	p-value
Intercept	1.385	0.4344	2.667	0.1316
MHDI	−3.292	0.3326	−4.820	0.1653
Deforestation area log	0.608	0.0018*	0.600	0.0035*
Moran’s I of residues	0.290	0.0000*	0.040	0.2554
Lambda	-	-	0.581	0.0005*
AIC	211.87	-	201.88	-

*Statistically significant difference, considering p-value < 0.05.

**Figure 3:**
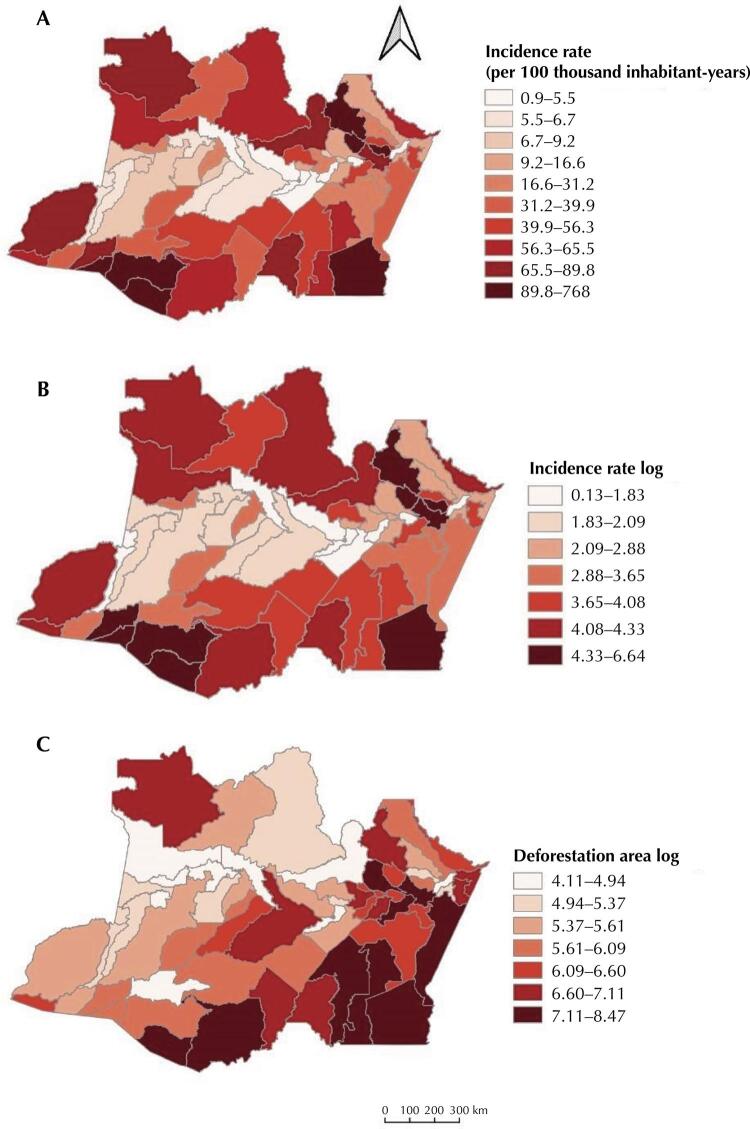
Incidence of American Cutaneous Leishmaniasis in the state of Amazonas. (A) Standardized incidence rates of American Cutaneous Leishmaniasis (per 100,000 population-years). (B) Log of the incidence rates used here as the response variable; (C) Log of the areas of deforestation in all 62 municipalities in the state of Amazonas, Brazil, from 2016 to 2020.

With the help of the spatial error model, it was found that the variations in one unit of the log of the deforested area from 2016 to 2020 are related, in the municipalities of Amazonas, to an increase of 0.600 in the log of the incidence rate per 100,000 inhabitants-year. Using exponentiation on the natural scale, a one unit rise in the log of the deforested area was equivalent to 1.82 cases of ACL per 100,000 residents. The highest degree of deforestation was observed in the municipalities of Lábrea, Boca do Acre, Apuí, Manicoré, Itacoatiara, Novo Aripuanã, Maués, Silves, and Manaus, municipalities which normally show high incidence values (Figure 3). The MHDI was not related to the ACL incidence rate.

**Figure 4:**
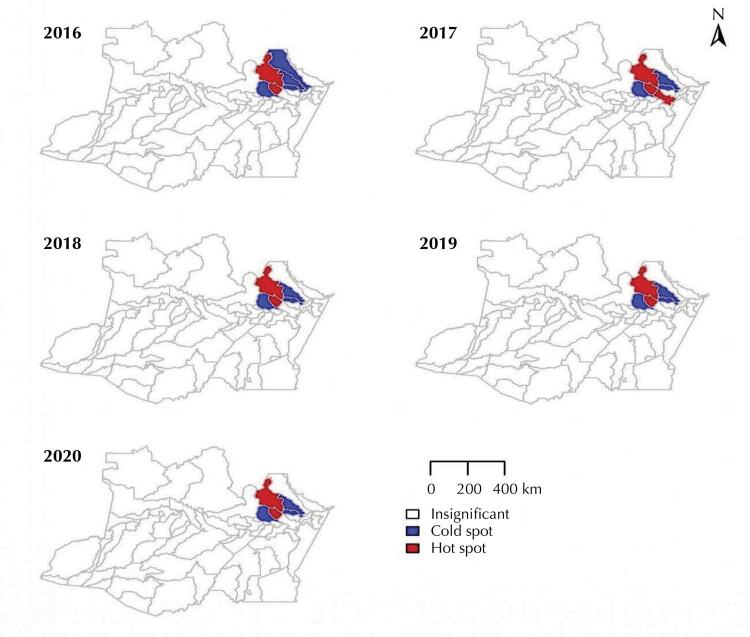
The Getis-OrdGi* statistic including the False Discovery Rate (FDR) adjustment for the municipalities in the state of Amazonas, Brazil, from 2016 to 2020. The municipalities shown in red (Presidente Figueiredo, Rio Preto da Eva, and Itacoatiara) indicate the hot spots for American Cutaneous Leishmaniasis, whereas those in blue (Manaus, Itapiranga, and São Sebastião do Uatumã) highlight the cold spots for American Cutaneous Leishmaniasis in the state.

The Getis-OrdGi* statistic, with the FDR corrections made for the significance level, helped to reveal the areas with high and low rates of ACL incidence. During the study period, two hotspots were observed near the Manaus region. The municipalities of Presidente Figueiredo and Rio Preto da Eva (Figure 4) displayed a high risk of ACL during the investigation period.

In 2017, Itacoatiara revealed a new hot spot, indicating that high-risk agglomerations increased that year. Cold spots, or sites with lower risk of disease, were found in the metropolitan area of the capital Manaus, Itapiranga, and São Sebastião do Uatumã, from 2016 to 2020, and in the municipality of Urucará only in 2016.

## DISCUSSION

In this study, the ACL incidence rates in all the municipalities in the state of Amazonas were quite heterogeneous and the disease showed a variety occurrence patterns in its spatial and temporal distribution. The municipalities of Rio Preto da Eva and Presidente Figueiredo recorded the highest incidence rates during the course of the study, from 2016 to 2020; these results concur with the findings of a previous study carried out by Rodrigues et al.^20^. Some of the municipalities in the state, including Rio Preto da Eva, showed high ACL incidence rates for several years, indicating the continued presence of the endemic disease.

This suggests the need for greater and more effective measures to reduce and control this disease and ensure better health for the population^20^. The complexity of the ACL epidemiology, with the related intra and interspecific variations in transmission cycles, leads to many different clinical manifestations and responses to treatment therapy^21^.

Regarding gender distribution, the findings of this study correspond to previous studies carried out in the state of Amazonas^10,22^, as well as in the other Brazilian states such as Pernambuco and Acre^11,23^. These studies reported a higher number of cases of the disease, mainly in males, as well as a greater frequency in the 20–39 age group, of mixed ethnicity, and among patients living mainly in rural regions^10,11,22,23^. Naiff Junior et al.^22^ suggested that the higher number of cases in males and young people might have something to do with work-related activities, such as agriculture. In fact, Tamponi et al.^9^, in their study on the occurrence of ACL using a multivariate analysis of the spatial production circuits in Minas Gerais, emphasized that those in closer contact with forests and those who work in rural areas are more likely to contract the disease because of the higher risk of exposure to the vector and its reservoirs.

The ACL shows the spatial distribution in the state of Amazonas in a heterogeneous pattern, revealing sites of higher and lower risk in space. The Getis-Ord Gi* statistic identified high-risk spatial clusters which highlighted the fact that particular attention is required from surveillance systems at municipal and state level to control ACL, especially in the municipalities of Presidente Figueiredo and Rio Preto da Eva, where the highest incidence rates and high risks for ACL were observed during the period of investigation. According to Figueira et al. ^24^, some municipalities have been rendered endemic for ACL, because of the agrarian settlement areas, as observed, for instance, along the Manaus-Itacoatiara highway (AM 010), in the municipalities of Rio Preto da Eva and Presidente Figueiredo (BR-174). Most of the patients with ACL who received treatment from the Tropical Medicine Foundation of Amazonas (FMT-AM), in Manaus, came from the vicinity of these regions, located along the highways and their branching side roads^24^.

The average number of ACL cases recorded were 1,499 per year, and some of the municipalities showed variations in the incidence rates, with upward trends over the five year period of the study, particularly the municipalities in the central part of the state, surrounding Manaus, as well as those in the southern region.

In light of the socio-environmental variables, the occurrence of ACL and the MHDI showed no significant relationship with each other; furthermore, these findings corresponded to the results of Guerra et al.^25^, in the Purus area, situated between the Amazonas and Acre states. An important result of this study is the finding of a positive association, with statistical significance, between the incidence of ACL and deforestation during the study period (2016 to 2020), suggesting that this is one of the factors that induced the permanence and rise in the incidence of ACL in Amazonas. Pérez-Flórez et al. ^26^ also reported a positive relationship between deforestation and ACL in Colombia, a country that borders the state of Amazonas.

For Franco^27^, the loss of habitat or decrease in vegetation could be linked to the BR-364 highway, which promotes the illegal transportation of timber and its removal in the areas to the south of the municipality of Lábrea. This highway took shape with the inclusion of several settlements, the addition of agricultural and mining projects, and confrontations between the native population and rubber tappers. Therefore, considering the negative effects associated with deforestation, the Brazilian government initiated a Land Regularization Program, in addition to other public policies, especially in the municipality of Lábrea^7^.

In their study, Peixoto^28^ showed that the variations in the prevalence of ACL cases may have been influenced and induced by the spurt in urbanization and by residences that encroach into forest areas that support and encourage the multiplication of vectors and reservoirs of the disease agents. Most cases in the state of Amazonas have been found in concentrated spots in deforested areas, especially on the outskirts of Manaus, as well as in the five main municipalities in the interior parts of the state: Rio Preto da Eva (AM-010), Presidente Figueiredo (BR-174), Itacoatiara (AM-010), Humaitá (BR-319), and Coari. In fact, Lonardoni et al. ^29^ observed large numbers of sandflies at home and in the neighboring areas, especially those in the northern regions of the state of Paraná, in a rural settlement in the Mariluz municipality. The authors related this to deforestation carried out to build houses. Therefore, the presence and continuity of ACL cases in Amazonas may be linked to urbanization, city growth and development, land invasion, road construction, and the construction of hydroelectric plants and waterways. This development is encouraged by public policies that promote the advance of agriculture in steady waves, starting in the states of Rondônia, Amazonas, and Acre and extending outwards^25^.

The limitations of this research must be acknowledged; they are due to the use of secondary data, with emphasis on the underreporting of ACL cases and the incomplete or completely ignored fields in the questionnaire forms, thus providing only partial information. To overcome limitations of this type, data cleansing was performed on the databases from the Health Surveillance Foundation of Amazonas, and on the IBGE’s geographic database. With this measure, the need to compensate for the inconsistency and incompleteness of the data in the items of their records, such as nominal adequacy of municipalities, special characteristics, and recognition of geographic aspects and details, was overcome.

Spatial analysis has gained importance as a reliable tool for identifying the various disease patterns that occur, thus helping in the proper and pertinent use and optimization of ACL control measures, as well as monitoring the continuity of these steps. This study, by applying the geoprocessing technique, was able to reveal the distribution of ACL in the state of Amazonas; also, the main risk areas and the related factors, such as deforestation, could be identified during the years of 2016 to 2020.

For designing and implementing epidemiological surveillance measures at the municipal and regional level, this type of data is crucial since it includes the prevalence patterns of ACL in each region, within high-risk regions. In addition to including the previous steps that were put on hold during the COVID-19 pandemic. Moreover, since deforestation has become a serious public health problem, affecting the transmission dynamics of different diseases associated with insect vectors, our results point to the need for public policies focused on environmental protection in this region. Our findings will provide valuable insights for public health decision-makers, since they clearly identify high-risk areas that require surveillance efforts toward ACL prevention and control.
